# Spatio-spectrally Tailored Multimode Metasurface Lasers in the Visible Range

**DOI:** 10.1021/acs.nanolett.5c06344

**Published:** 2026-03-20

**Authors:** Ayesheh Bashiri, Aleksandr Vaskin, Katsuya Tanaka, Muyi Yang, Thomas Pertsch, Isabelle Staude

**Affiliations:** † Institute of Solid-State Physics, Abbe Center of Photonics, 9378 Friedrich Schiller University, Jena 07743, Germany; ‡ Institute of Applied Physics, Abbe Center of Photonics, 9378 Friedrich Schiller University, Jena 07745, Germany; § Fraunhofer-Institute of Applied Optics and Precision Engineering IOF, Jena 07745, Germany; ∥ Max Planck School of Photonics, Jena 07745, Germany

**Keywords:** dielectric metasurfaces, lasing, bound states in the continuum, guided-mode resonances, surface lattice resonance

## Abstract

Spectrally engineered multifrequency nanolasers are highly desirable for on-chip photonics, multiplexed biosensing, and display technologies, yet achieving them within a compact platform remains challenging. Here, we demonstrate multimode lasing from symmetry-broken TiO_2_ metasurfaces integrated with an SU8 slab waveguide containing rhodamine 6G. By coengineering guided-mode resonances, surface lattice resonances near Rayleigh anomalies, and quasi-bound states in the continuum, we realize complementary high-Q feedback pathways that overlap with the gain spectrum. The lasing emission direction is tailored through outcoupling via second-order Bragg diffraction and Rayleigh anomaly conditions, supporting both normal and oblique emission. Experiments reveal discrete lasing outputs across ≈100 nm bandwidth (548–648 nm), spanning the full rhodamine 6G emission band, with thresholds as low as ∼7 nJ (35.7 μJ/cm^2^) and up to four concurrent lasing peaks from a single device. These results establish a metasurface-dye platform for multifrequency and angle-selective lasing, opening new opportunities for compact, multifunctional nanophotonic sources.

## Graphical abstract

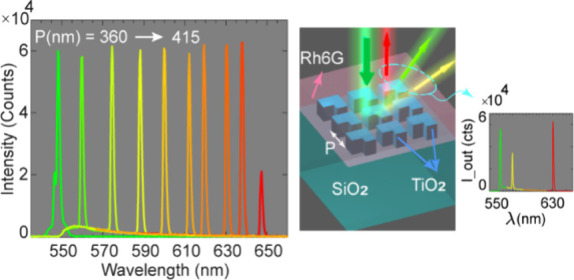

Compact laser sources capable of multiwavelength emission with controllable directionality are highly desirable for next-generation photonic technologies.^1–3^ Realizing such functionality within a single planar architecture requires multiple optical resonances with distinct spectral and angular characteristics, strong light confinement, and minimal losses. All-dielectric metasurfaces, composed of periodic arrays of high-index nanostructures, are well suited for this purpose because they can host a rich set of high-quality (Q)-factor resonances in the visible with far lower intrinsic loss than plasmonic counterparts.^4–9,11,26–30^ Combined with subwavelength thickness and geometric tunability, dielectric metasurfaces, when integrated with an efficient gain medium, provide an ideal route to spectrally and directionally engineered multifrequency lasing within a compact footprint. Low-threshold operation, however, requires more than simply minimizing loss. The lasing mode must also exhibit strong spatial and spectral overlap with the gain medium to maximize light–matter interaction.^10,11^

In metasurfaces cladded with a gain layer, several routes can supply the required optical feedback. A widely used pathway is diffraction-coupled band-edge lasing, in which the lattice couples in-plane propagating modes to the far-field at specific points of the dispersion.^12,13^ Such band edges can originate from guided-mode resonances (GMRs),^14,15^ in which waveguide-like modes in the gain layer couple to the lattice through Bragg scattering,^16,17^ or from surface lattice resonances (SLRs),^18–21,30,31^ namely collective modes arising from the diffractive coupling of particle resonances near the Rayleigh anomaly (RA). At the band edge, the group velocity approaches zero, resulting in strong field localization and an enhanced Q-factor. Under the second-order Bragg condition, these modes couple efficiently to the zeroth diffraction order, enabling normal-direction emission, while satisfying the Bragg condition for other diffraction orders allows lasing at defined angles.

Another pathway is through bound states in the continuum (BICs), which are modes lying above the light line yet completely decoupled from radiative channels. In dielectric metasurfaces, BICs typically arise when the mode field distribution possesses a symmetry (e.g., even/odd parity) incompatible with that of a plane wave at normal incidence, thereby preventing out-of-plane coupling. Introducing a controlled asymmetry, such as a geometric perturbation, converts the ideal BIC into a quasi-BIC, where the radiative leakage becomes finite but can be tailored to remain very small. This enables outcoupling in the normal direction while preserving the high Q-factor required for efficient optical feedback.^22–27^

Each of these routes has been leveraged separately for lasing. Azzam et al. reported near-Γ, BIC-assisted lasing from (titanium dioxide) TiO_2_ nanoresonator arrays via lattice–dipole coupling, achieving single and multimode lasing at small off-normal angles.^28^ Low-threshold lasing was enabled by high-Q, symmetry-protected BICs originating from first- and second-order transverse-electric (TE)-polarized slab-waveguide modes coupled to the periodic array of TiO_2_ nanoantennas.^11^ Barth et al. achieved lasing from a large-area 2D material using a metasurface supporting both quasi-BIC and GMR modes, enabling Γ-point lasing with similar thresholds.^29^ Yang et al. demonstrated low-threshold Γ-point lasing in hybrid SLR Si_3_N_4_ metasurfaces by deliberately breaking structural symmetry to convert ideal BICs into quasi-BICs.^30^ In a related plasmonic structure, Guan et al. realized white-light lasing by sandwiching three different dye solutions between metasurfaces of different periods, using multimodal SLRs to generate RGB lines with tunable relative intensities, underscoring spectral multiplexing within a single device.^31^ Despite these advances, coengineering multiple mode classes within one device, specifically, SLRs, GMRs, and quasi-BICs, to achieve simultaneous multiwavelength and multiangle lasing remains unexplored.

Here, we present symmetry-broken all-dielectric metasurfaces composed of L-shaped^32,33^ TiO_2_ nanoresonators (*n* ≈ 2.4) on a silicon dioxide (SiO_2_) substrate (*n* ≈ 1.45). The metasurfaces are spin-coated with an SU8 layer (*n* ≈ 1.6) doped with rhodamine 6G (Rh6G) laser dye (Figure 1a). TiO_2_ combines a high refractive index with negligible absorption across the visible spectral range. Rh6G offers a broad gain spectrum, high quantum yield, and ease of integration.^34,35^ With air above and SiO_2_ below, the SU8 core forms an asymmetric slab waveguide, while the metasurface at the SU8-SiO_2_ boundary plays a dual role: it supports localized multipolar resonances that, under intentional symmetry breaking, evolve into quasi-BICs, while its periodicity provides the reciprocal-lattice momentum needed for SLR formation, GMR coupling, and second-order Bragg feedback at band edges, enabling vertical outcoupling at Γ.

**Figure 1:**
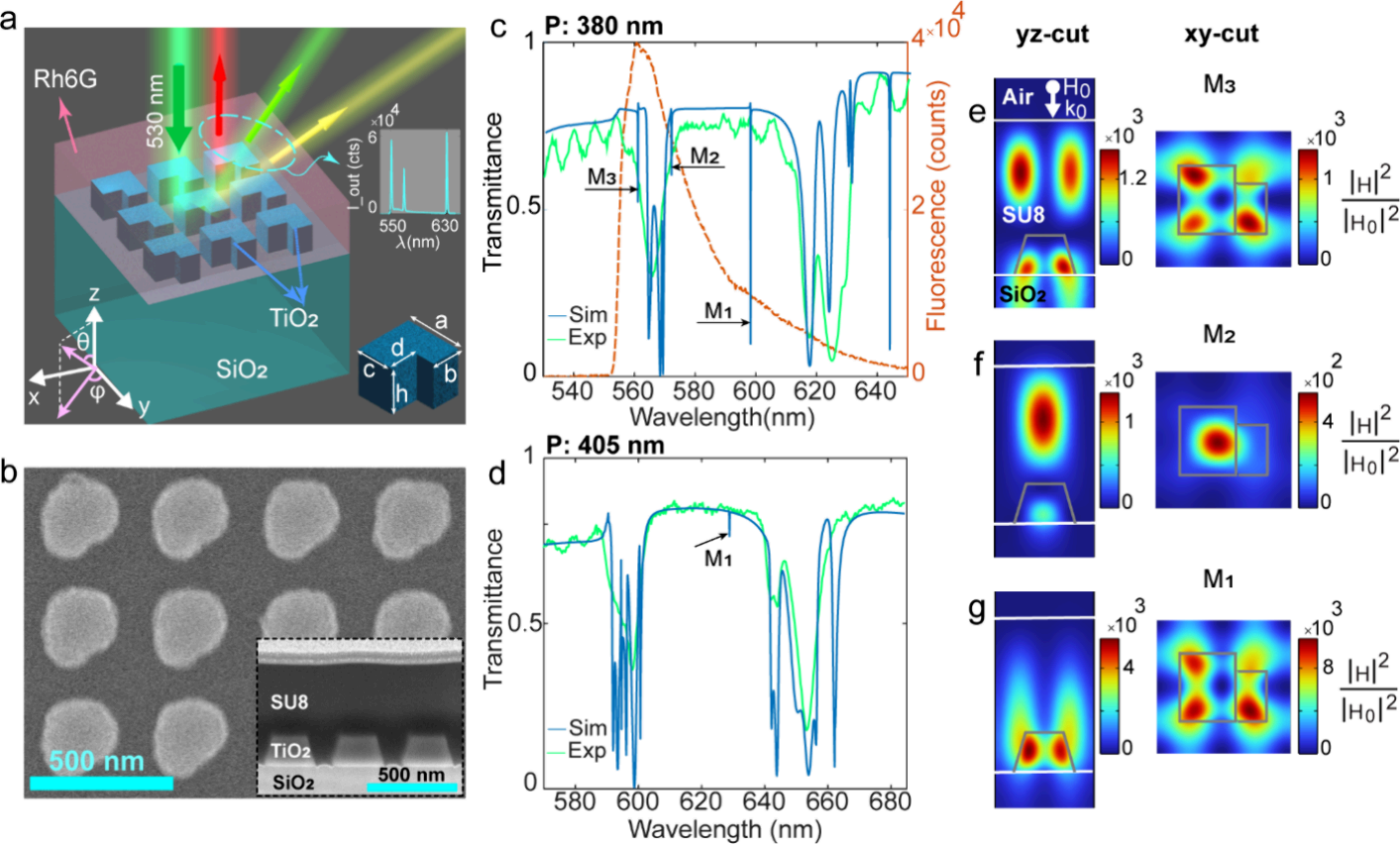
(a) Schematic illustration of lasing metasurface showing an array of L-shaped TiO_2_ nanoresonators on a SiO_2_ substrate, integrated with an Rh6G-doped SU8. Under a 532 nm pumping, the coupled system generates triple-mode lasing at 550 nm (green), 570 nm (yellow), and 630 nm (red). (b) Top-view scanning-electron micrograph (SEM) of the fabricated metasurface with a period of 380 nm. The inset shows a focused-ion-beam cross-section SEM of the metasurface coated with Rh6G-doped SU8 of 570 nm thickness. (c, d) Measured and numerically calculated linear optical transmission spectra of the coated metasurfaces with a period of (c) 380 nm (LMS1) and (d) 405 nm (LMS2), for *y*-polarized normal incidence illumination. The high-Q modes leading to lasing at normal incidence for LMS1 are denoted with M_1_–M_3_. The red dashed line in (c) represents the fluorescence spectrum of Rh6G measured with a 550 nm long-pass filter. (e–g) Calculated near-field intensity profiles of LMS1 for the modes M_1_–M_3_ in the cross-section through the center of the nanoresonator for *y*-polarized normal incidence illumination. All mode profiles are normalized with respect to the intensity of the incident plane wave. The nanoresonator outline is indicated with gray solid lines.

Through careful control of slab thickness, lattice period (*P*), and asymmetry, this platform brings GMRs, SLRs, and quasi-BICs into deliberate coexistence. Leveraging multiple mode types is essential to achieve broad spectral coverage for lasing within a single dye-integrated metasurface. Simply increasing the waveguide thickness to support higher-order GMRs sacrifices compactness and typically weakens their phase-matched coupling to the lattice and the vertical channel at the Γ-point. SLRs, in contrast, arise only at discrete band edges defined by diffraction orders (Rayleigh conditions), leading to relatively large free spectral ranges for a given period. Quasi-BICs provide high Q-factor resonances, but their fields remain concentrated largely within the high-index resonators, limiting overlap with the dye unless hybridized with lattice or guided modes.

By combining modes of different physical origins, our platform provides complementary resonances with tunable spacing and coupling efficiency, enabling multiple spectrally engineered lasing peaks across the Rh6G gain bandwidth. Depending on the design, emission is directed either along the surface normal or into oblique angles, unifying spectral and directional control within a single metasurface, an advance beyond previous multimode lasing systems.

Experimentally, we demonstrate lasing across the full Rh6G gain bandwidth, spanning from 548 nm (green) to 648 nm (red). To the best of our knowledge, the 548 nm lasing represents the shortest-wavelength laser reported for Rh6G, while the 648 nm peak extends to the dye’s longest accessible wavelength. Depending on the structural design, we observe up to four simultaneous lasing peaks, all with relatively low thresholds on the order of ∼ 7 nJ (35.7 μJ/cm^2^) incident pump pulse energy (PPE). For a suitably optimized metasurface design, lasing peaks are observed at 550, 570, and 630 nm, corresponding to green, yellow, and red emission, respectively.

Our approach demonstrates passive spectral and angular multiplexing in a monolithic, all-dielectric platform, without the need for active tuning or multiple gain materials. This work establishes a new paradigm for multifunctional nanophotonic sources with potential impact on next-generation optical communication, sensing, and display technologies.

For the design of the multifrequency metasurface lasers, we started by selecting *P* so that the first-order RAs (λ_RA_ = *n* × *P*) fell within the Rh6G gain bandwidth (Figure 1c), yielding an SLR near λ = 550 nm from the SiO_2_ substrate and another near λ = 600 nm from the SU8 cladding. Next, using finite-element simulations (COMSOL Multiphysics), we designed a square lattice of cubic nanoresonators with a small notch in the corner to break in-plane symmetry and generate high-Q quasi-BICs within the spectral gain bandwidth.

We calculated normal-incidence transmission under x- and y-polarized illumination (Section S1). We performed parametric sweeps of the SU8 thickness to tune the resonances of the lowest-order GMRs into the Rh6G gain bandwidth, while varying the resonator dimensions and notch depth to achieve the same for the quasi-BIC mode. The resulting spectra for y-polarized incident light and for lasing metasurfaces with *P* = 380 nm (LMS1) and 405 nm (LMS2) are shown in Figure 1c,d, respectively. Corresponding spectra for x-polarized incident light are shown in Figure S1. The geometrical parameters according to the inset of Figure 1a are listed in Table 1. For LMS1, three sharp high-Q modes (Q-factors in Supporting Information (SI)), labeled as M_1_ at 597 nm, M_2_ at 571 nm, and M_3_ at 560 nm, fall within the Rh6G gain bandwidth. In LMS2, all resonances redshift with M_1_ appearing at 630 nm.

**Table 1:** Designed and Experimentally Realized Geometrical Parameters for LMS1 and LMS2

		Parameters^a^ (nm)					
		*a*	*b*	*c*	*d*	*h*	SU8 height
LMS1(*P* = 380)	Sim	142	119	105	79	153	593
	Fab ≈	162	113	102	67	156	570
LMS2(*P* = 405)	Sim	175	81	110	75	153	593
	Fab ≈	162	113	102	67	156	570

a See the inset of Figure 1a for the definitions.

We performed high-resolution reciprocity-based^37^ angle-resolved dispersion mapping near Γ for symmetric and broken-symmetry geometries, along with an asymmetry-variation study (Section S3, S13). In the symmetric case, M_1_ and M_2_ are strongly suppressed in the far field (with M_3_ weakly visible) and become radiative only with intentional symmetry-breaking, consistent with high-Q quasi-BIC. We then calculated near-field intensity cross sections through the resonator center for M_1_–M_3_ (Figure 1e–g, Figure S3a–c) and performed mode-overlap analysis (Section S4) and multipole decomposition (Section S5). The results show M_1_ (600 nm) is dominated by an in-plane magnetic quadrupole (MQ), which is intrinsically symmetry-protected. The field is strongly concentrated in the TiO_2_ nanoresonators but extends into the SU8 slab waveguide, enabling overlap with the gain medium. The *yz*-cut shows a single-lobed E_*z*_-dominant distribution (Figure 1g, S3c) resembling a transverse-magnetic (TM)_0_-like GMR.^18^ M_2_ (570 nm) is dominated by an out-of-plane magnetic dipole (MD_*z*_), likewise dark in the symmetric case. Its field profiles reveal two vertically separated antinodes with a central node and a dominant H_*z*_ component (Figure 1f, S3b), matching the TE_1_-like GMR in the SU8. Finally, M_3_ (560 nm) is dominated by the same in-plane MQ mode as M_1_. The yz-cut shows two antinodes separated by a node along z with a dominant E_*z*_ component (Figure 1e, S3a), resembling a TM_1_-like GMR in the SU8 slab, while spectral proximity to the SiO_2_-side RA and substrate-extended fields indicate SLR hybridization.^18,36^

Using the optimized design parameters for the nanoresonators and SU8 thickness, we fabricated metasurfaces with periods ranging from 330 to 415 nm using electron beam lithography followed by reactive ion etching (see SI). To compensate for fabrication tolerances, the lateral dimensions of the nanoresonators were varied by adjusting the exposure dose to ensure accurate realization of the designed structures. Figure 1b shows a scanning electron micrograph (SEM) of LMS1, exhibiting a slight corner rounding; otherwise, the dimensions closely match the design (Table 1).

Subsequently, the fabricated metasurfaces were spin-coated with a 570 nm-thick SU8 layer doped with Rh6G dye (Section S7). A focused-ion-beam cross-section of the coated metasurface is shown in Figure 1b, inset. Nanoresonator sidewalls exhibit a slope of ≈70° relative to the substrate, as a consequence of the etching process. This feature was incorporated in all design and simulation steps to ensure accurate performance modeling.

To precharacterize the coated metasurfaces, we measured polarization-resolved, normal-incidence transmission for LMS1 and LMS2. The measured spectra for y-polarized and x-polarized incident light are shown in Figure 1c,d, and S1a,b. A good qualitative agreement with numerically calculated spectra is observed, considering that the numerically predicted high-Q resonances are not resolved due to finite angular collection (NA ≈ 0.04, ±2.5°) and the spectrometer resolution (full-width at half-maximum (FWHM) = 1.34 nm). However, these modes become evident under optical pumping as lasing peaks and are further confirmed through far-field emission characterization.

To experimentally characterize the lasing from the dye-integrated metasurfaces, we performed power-dependent fluorescence spectroscopy. To this end, we pumped metasurfaces using a 532 nm pulsed laser (0.5 ns pulse duration, 1 Hz repetition rate) over a PPE range of 0–9 nJ (0–45.9 μJ/cm^2^), with each spectrum integrated over a 1 s acquisition period. The collimated beam passed a square aperture, which was imaged onto the selected metasurface to provide uniform illumination confined to the metasurface patch, with a square spot size of 140 μm per side. Emission was collected with a 0.9-NA objective (polar angle θ = ±64°) and directed either to a spectrometer or to a camera imaging the back-focal plane (BFP) of the objective (see SI). Figure 2a presents power-dependent emission from LMS1 with increasing PPE. At 6.7 nJ, the spectrum follows the broad Rh6G fluorescence (spontaneous emission). At 6.9 nJ, a narrow line appears at 600 nm, consistent with M_1_ in Figure 1c. With further increase (≈7.6 and 8.4 nJ), additional peaks emerge at 574 nm (M_2_) and 559 nm (M_3_). Figure 2b exhibits output intensity (*I*_out_) (blue) and FWHM (orange) as functions of PPE. The former exhibits the characteristic S-curve for all three peaks, with clear thresholds of ∼6.9–8.4 nJ PPE (35.2–42.9 μJ/cm^2^), confirming lasing at 600, 574, and 559 nm. Simultaneously, the FWHM narrows down from ≈30 nm to ≈0.26 nm (see SI for line width extraction method).

**Figure 2:**
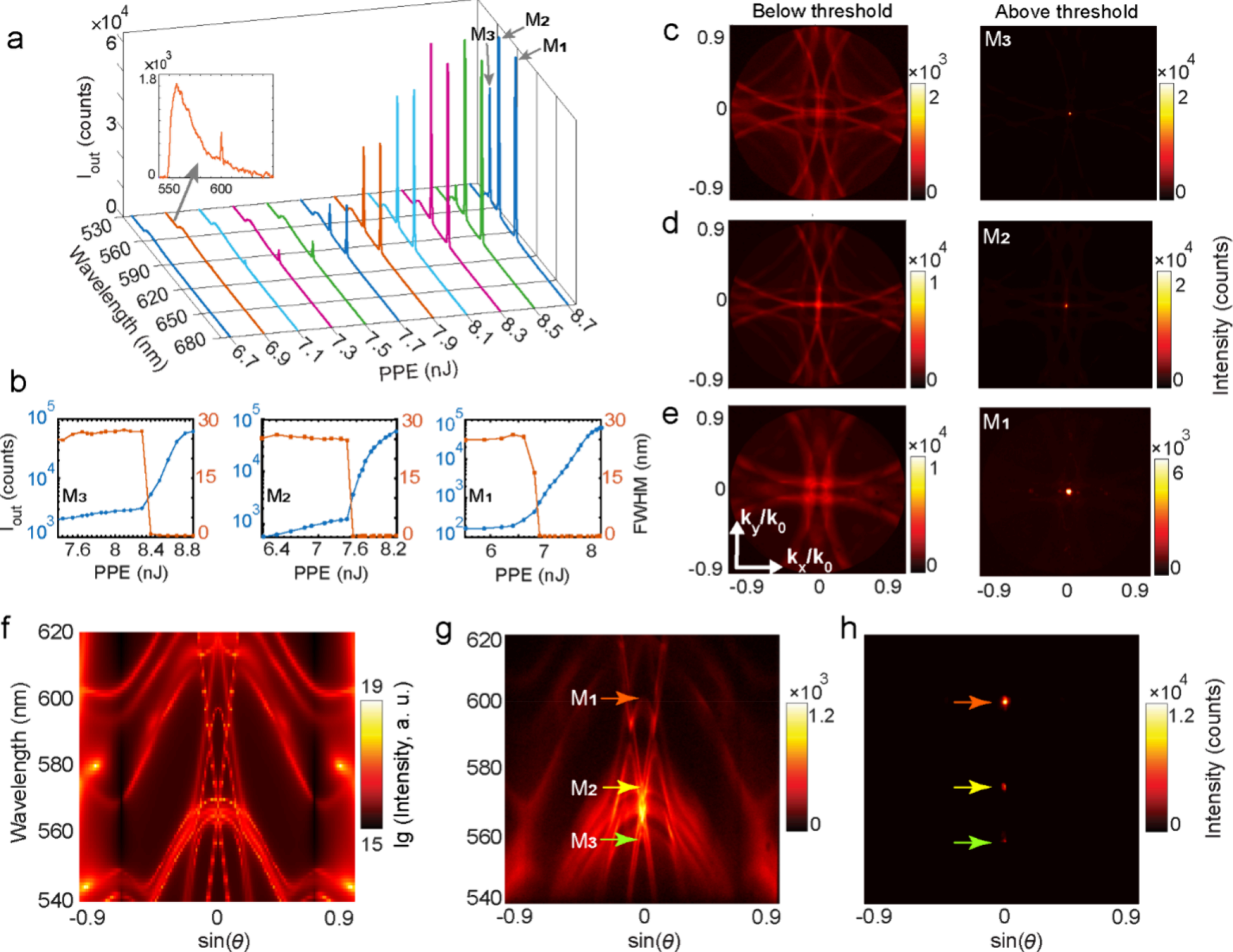
(a) Evolution of the emission spectra of LMS1 at different PPEs. The inset shows the initial emergence of the lasing peak associated with resonance M_1_ at 6.9 nJ PPE. (b) Maximum output intensity *I*_out_ at different PPEs (S-curves (blue) on log–log scale (see SI for linear–linear scale) and FWHM (orange) for M_3_–M_1_. (c–e) Measured BFP images of LMS1 using band-pass filters centered at (c) 560 nm (M_3_), (d) 570 nm (M_2_), and (e) 600 nm (M_1_). Below threshold (left) and with a repetition rate of 500 Hz and an integration time of 2 s for improved signal strength, it shows the characteristics of spontaneous emission into the spatially dispersive metasurface modes, dominated by diffractive features. Above threshold (right) at PPE (1 Hz, 1 s) (c) 8.6 nJ, (d) 7.9 nJ, and (e) 6.1 nJ, lasing emission becomes dominant, manifesting as a bright, sharp feature in the center, corresponding to beaming. (f) Numerically calculated angular resolved spectrum for an angle φ = 0 and θ up to ± 64° averaged over TE and TM polarization of the incident light. (g, h) Measured momentum-resolved spectra below (g) and above (h) threshold.

Polarization-dependent measurements are presented and discussed in Section S12. Furthermore, an eigenfrequency calculation with a small artificial gain in the SU8 layer confirmed that modes M_1_–M_3_ are the first to acquire net amplification upon increasing the gain, consistent with the experimentally observed lasing peaks (SI).

Next, we characterized the far-field emission of LMS1 using BFP imaging (Section S10) with band-pass (BP) filters (10 nm bandwidth) centered at 560, 570, and 600 nm (Figure 2c–e). Note that these images are isofrequency slices of the angular dispersion, rather than the exact lasing ridge. For example, M_2_ lases at ≈574 nm; however, the 570 nm filter represents the nearby band edge features. Below threshold (left), the images display the characteristic diffraction circles of the periodic lattice, consistent with spontaneous emission coupled into the lattice modes. Above threshold, the emission at all three wavelengths concentrates into a sharp spot at the center of the corresponding BFP images (*k*_*x*_,*k*_*y*_ = 0), where (*k*_*x*_,*k*_*y*_) are the in-plane components of the photon momentum, demonstrating normal-direction outcoupling of the lasing emission.

To further characterize the lasing emission, we performed momentum-resolved spectroscopy by spectrally resolving a thin slice of the BFP images around *k*_*y*_ = 0 (azimuthal angle φ = 0). The results (Figure 2g) show radiative branches with two pronounced Γ-centered band edges near ∼600 nm and ∼570 nm, evident as dispersion flattening and imposed by the lattice periodicity under the second-order Bragg condition. M_1_ lies at the ∼600 nm band edge, while M_2_ and M_3_ are associated with the upper and lower edges of the ∼570 nm band, respectively. The measured dispersion is reproduced by reciprocity-based simulations (Figure 2f, Section S13). To relate these bands to the available radiation channels, we overlaid RA diffraction curves for the SU8 and the SiO_2_ sides (see Section S3, Figure S2a). The Γ-centered band edges lie in the vicinity of these diffraction channels, consistent with the hybrid Bloch modes character inferred from the near-field and multipolar analyses.

Next, to investigate lasing characteristics in LMS2 (*P* = 405 nm), we repeat all the measurements starting from power-dependent fluorescence spectroscopy. As shown in Figure 3a, three peaks emerge at 550, 570, and 630 nm with close threshold values of ≈7.8 nJ (39.8 μJ/cm^2^) for the first two and ≈7 nJ (35.7 μJ/cm^2^) for the latter.

**Figure 3:**
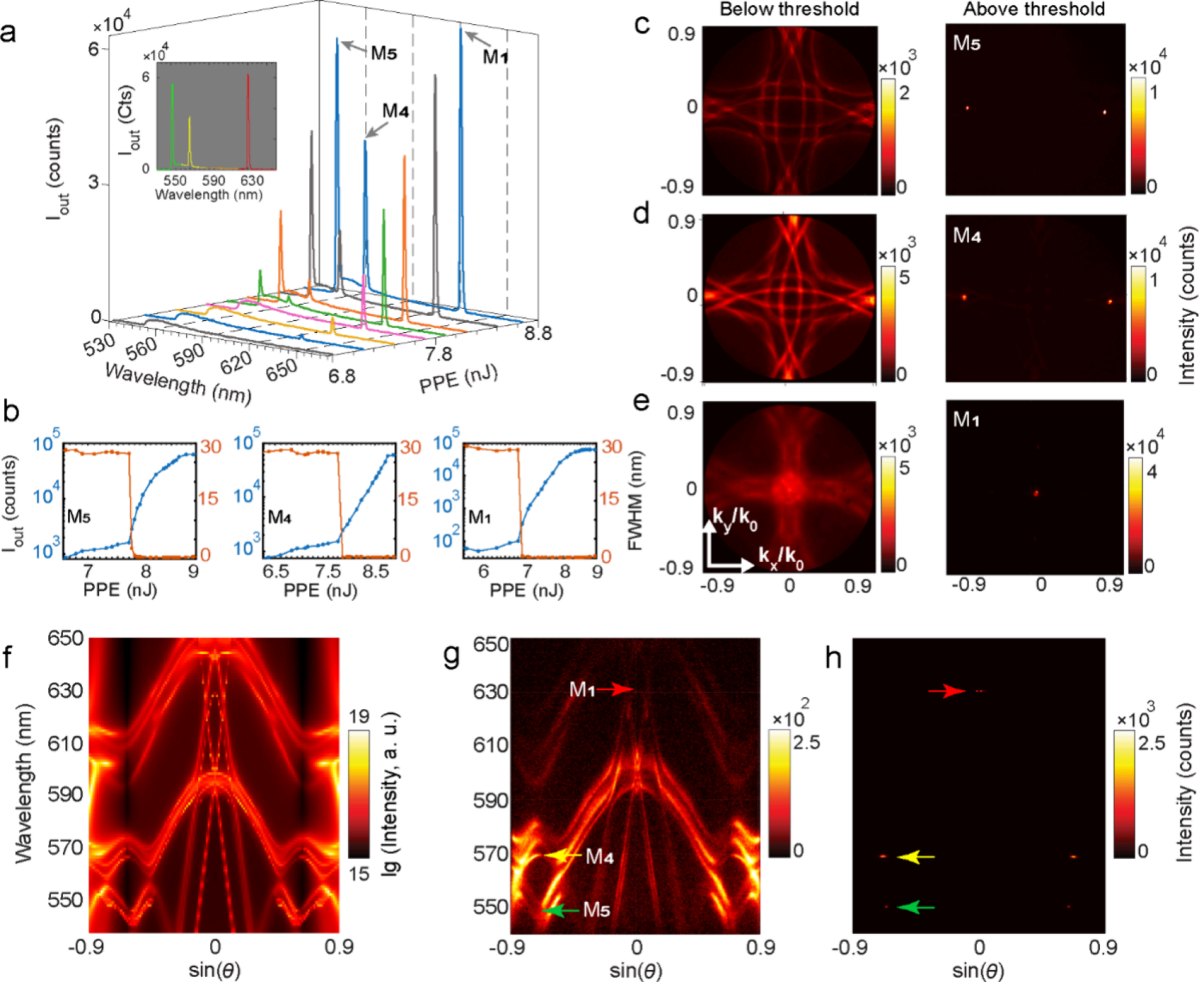
(a) Evolution of the emission spectra of LMS2 at different PPE. The inset shows the lasing spectrum (at 8.7 nJ) with discrete-frequency outputs ranging from green to red. (b) Maximum output intensity *I*_out_ (blue) and FWHM’s (orange) at different PPEs for M_5_, M_4_, and M_1_. (c–e) Measured BFP images of LMS2 using BP filters centered at (c) 550 nm (M_5_) and (d) 570 nm (M_4_), as well as (e) an LP filter at 610 nm (M_1_). Below the threshold (left) and with a repetition rate of 500 Hz and an integration time of 2 s to increase the signal strength, spontaneous emission occurs in the shape of broad diffraction rings from the periodic array. Above threshold (right) at PPE (1 Hz, 1 s) (c) 8 nJ, (d) 8.2 nJ, and (e) 8 nJ, lasing emission becomes dominant under θ ≈ ±42° for M_5_, θ ≈ ±45° for M_4_, and near normal direction for M_1_. (f) Numerically calculated angular resolved spectrum and (g, h) measured momentum-resolved spectra below (g) and above (h) threshold.

The shorter-wavelength modes, denoted as M_4_ and M_5_, as described in the next section, are new resonances, while the 630 nm peak is the red-shifted counterpart of M_1_ from LMS1. In the inset (8.7 nJ PPE), we can clearly see that the spectrum spans green to red, demonstrating an exceptionally broad band for a single-dye lasing metasurface platform. The S-curves and power-dependent FWHM evolutions (Figure 3b) confirm lasing for all three modes. Figure 3c–e shows BFP images for the three lasing modes M_5_, M_4_, and M_1_, measured with BP filters at 550 and 570 nm and a long-pass (LP) filter at 610 nm, respectively. Below threshold (left), the patterns show the characteristic spontaneous emission of periodic metasurfaces, appearing as broad diffraction rings from the nanoresonator lattice. For M_5_ (550 nm) and M_4_ (570 nm), no Γ-point states are present (Figure 3c,d). In contrast, for M_1_ (630 nm), the band-crossings at Γ remain evident, consistent with normal-direction lattice-mediated outcoupling. Note that the features appear slightly broadened for M_1_ because we used an LP rather than a BP filter. Above threshold (right), the emission condenses into bright spots: at θ ≈ ± 42° for M_5_, θ ≈ ± 45° for M_4_, and near-normal direction for M_1_.

Figure 3g,h shows momentum-resolved spectra below and above threshold: dispersive radiative branches appear around 630 nm and form a Γ-centered band-edge feature. The emission is weak as it lies at the tail of the Rh6G gain spectrum. Above threshold, a bright near-normal lasing peak emerges at 630 nm (M_1_) along with two oblique-angle lasing modes: M_5_ (550 nm) at θ ≈ ±42°, and M_4_ (570 nm) at θ ≈ ±45°, matching the below threshold band edges at these points. From the simulated angular-resolved dispersion (Figure 3f) with overlaid RA references (Section S3) and the calculated near-field profiles (Figure S8), we assign M_4_ to an SU8-side RA (SLR-dominated) and M_5_ to a GMR. The RA/GMR reference relations provide useful design guidance, linking period, wavelength, and emission angle to the relevant refractive/effective index. However, the exact quantitative agreement is not expected since the modes are hybridized, the relevant diffraction channels are defined by a finite SU8 cladding with the embedded metasurface rather than an ideal homogeneous half-space, and the GMR dispersion and coupling are also modified by strong scattering from the TiO_2_ nanoresonators.^18^

Finally, we systematically investigated metasurfaces with the same nanoresonator size and *P* ranging from 330 to 415 nm. Figure 4a illustrates the lasing spectra, where the main modes (M_1_–M_5_, indicated by colored lines) redshift with increasing period. As summarized in Figure 4b, this geometrical tunability enables tailoring of the emission wavelength(s) over a broad spectral range from 548 to 648 nm, spanning the entire Rh6G gain bandwidth, with comparable thresholds. Notably, 548 nm (*P* = 360 nm) and 648 nm (*P* = 415 nm) represent the shortest and longest lasing wavelengths reported for Rh6G-integrated metasurfaces (Figure S9). In addition to the single-, double-, and triple-mode operation shown in Figure 4a, we observe up to four simultaneous lasing peaks from a single metasurface design with larger nanoresonator lateral dimensions (Figure S10).

**Figure 4:**
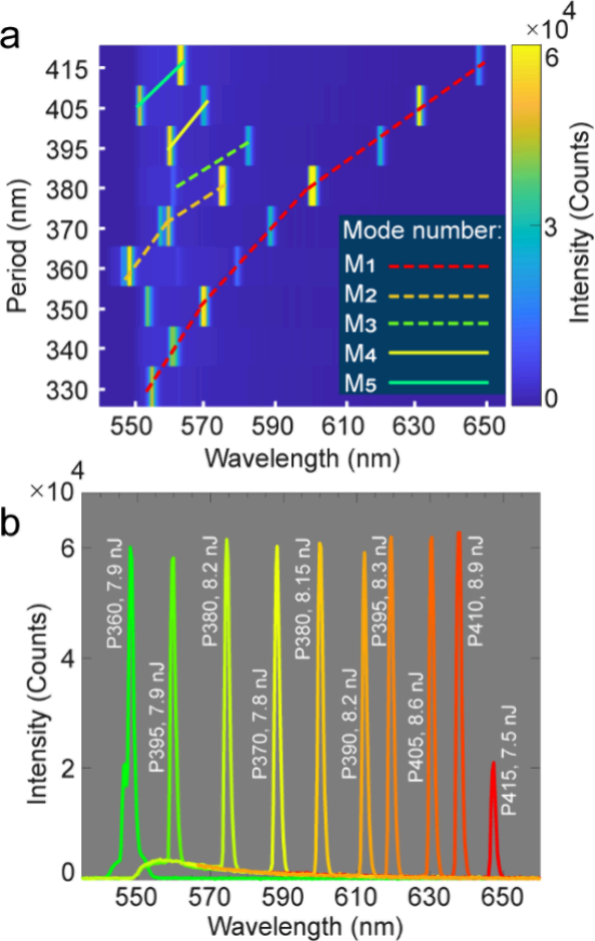
(a) Lasing emission spectra for metasurface periods ranging from 330 to 415 nm. The modes contributing to lasing M_1_–M_5_ are shown by colored lines. (b) Spectral tunability of the lasing emission, supported by metasurfaces with different periods, is presented.

In summary, we demonstrate a Rh6G-integrated TiO_2_ metasurface laser that combines several complementary resonance types—GMRs, SLRs, and quasi-BICs—within a single device. By leveraging SLRs and GMRs whose spectral positions are primarily set by the lattice period and SU8 thickness, the lasing response remains robust against moderate geometric deviations such as corner rounding. The deliberate use of symmetry breaking and lattice design allows for tailoring a set of high-Q resonances with strong mode-gain overlap, enabling multifrequency lasing throughout 548–648 nm, including near the spectral limits of Rh6G. Outcoupling via second-order Bragg and Rayleigh conditions allows for tailoring the direction of the laser beam (normal and oblique), with consistent thresholds around ∼7 nJ PPE (35.7 μJ/cm^2^). Importantly, our approach demonstrates not only broadband coverage but also robust multimode operation, with up to four lasing peaks achievable within a single metasurface. This work offers a versatile route to engineer spectral and modal content as well as the beaming direction in compact nanolasers, with potential applications in integrated photonics, multiplexed sensing, optical communications, and advanced display technologies.^38–41^

## Supplementary Material



## Notes

Calculation of transmission spectra, q-factor of the modes in LMS1, mode analysis using angular resolved dispersion map, calculated normalized near-field intensity profiles and mode-overlap analysis, multipole decomposition of the modes, fabrication process of the metasurfaces, gain medium preparation, instrument-response-corrected line width extraction, S-curves on linear–linear scale, experimental setup, identifying lasing modes of LMS1 via eigenfrequency analysis, polarization-dependent emission spectra, calculation of the angle-resolved emission spectra, calculated normalized near-field intensity profiles of modes at an oblique angle, angular resolved emission spectra of lasing metasurfaces with the longest and shortest lasing wavelengths for Rh6G dyes, angular resolved emission spectra of the lasing metasurface with four lasing peaks, amplified spontaneous emission or lasing (PDF)

## Abbreviations

Q-factor: quality-factor
GMR: guided-mode resonance
SLR: surface lattice resonance
RA: Rayleigh anomaly
BIC: bound states in the continuum
TiO_2_: titanium dioxide
TE: transverse-electric
SiO_2_: silicon dioxide
Rh6G: rhodamine 6G
*P*: lattice period
PPE: incident pump pulse energy
LMS: lasing metasurface
MQ: magnetic quadrupole
TM: transverse-magnetic
MD: magnetic dipole
SI: Supporting Information
SEM: scanning electron microscopy
FWHM: full-width at half-maximum
BFP: back-focal plane
BP: band-pass
LP: long-pass.
