# Supplementary material for: Spatio-spectrally Tailored Multimode Metasurface Lasers in the Visible Range
Source: Nano Lett. 2026 Mar 20;26(12):4111–8. doi: 10.1021/acs.nanolett.5c06344 (PMC13047725; doi:10.1021/acs.nanolett.5c06344)
Supplement: Supplementary file 1 [file nl5c06344_si_001.pdf]

Supporting information for:
Spatio-spectrally tailored multimode metasurface lasers in the visible range

Ayesheh Bashiri,[*,†,‡] Aleksandr Vaskin,[‡] Katsuya Tanaka,[†,‡,∥] Muyi Yang,[†,‡,∥] Thomas Pertsch,[†,‡,∥] and Isabelle Staude,[†,‡,∥]

†Institute of Solid-State Physics, Abbe Center of Photonics, Friedrich Schiller University, Jena, 07743, Germany.

‡Institute of Applied Physics, Abbe Center of Photonics, Friedrich Schiller University, Jena, 07745, Germany.

§Fraunhofer-Institute of Applied Optics and Precision Engineering IOF, Jena, 07745, Germany.

∥Max Planck School of Photonics, Jena, 07745, Germany.

*Corresponding author. Email: Ayesheh.bashiri@uni-jena.de

S1: Calculation of transmission spectra

We calculated the transmission spectra of the metasurfaces using finite-element simulations (COMSOL Multiphysics) under normal incident x- and y-polarized illumination. An elementary unit cell with Floquet periodic boundary conditions and two ports (top and bottom) was employed. A normally incident plane wave was launched from the top port, and the reflected, transmitted, and diffracted fields were collected at both ports. The refractive index data of TiO_2 thin film used in the nanofabrication of this sample are provided in Ref [33] of the manuscript. As noted in the manuscript, while the measured transmission spectra are in good qualitative agreement with simulations, the sharp resonances are not resolved in the experiment. This discrepancy mainly reflects measurement-induced broadening and angular averaging rather than the absence of high-Q resonances. The simulations in Figure 1c,d, and Figure S1a,b correspond to ideal plane-wave illumination at a single incidence angle (normal incidence), whereas our transmission measurements have a finite angular acceptance (NA $\approx$ 0.04, i.e., $\approx \pm 2.5°$). For the strongly dispersive/symmetry-sensitive modes, averaging over only a few degrees significantly affects the resonance linewidth. In addition, the finite spectrometer resolution further broadens and shallows narrow features. We illustrate the angular-averaging effect in Figure S1c by simulating the y-polarized transmission of LMS1 over a representative spectral window around M_3 and averaging the spectra over incidence angles from 0–2.5°, which reproduces the observed weakening of the resonance features.

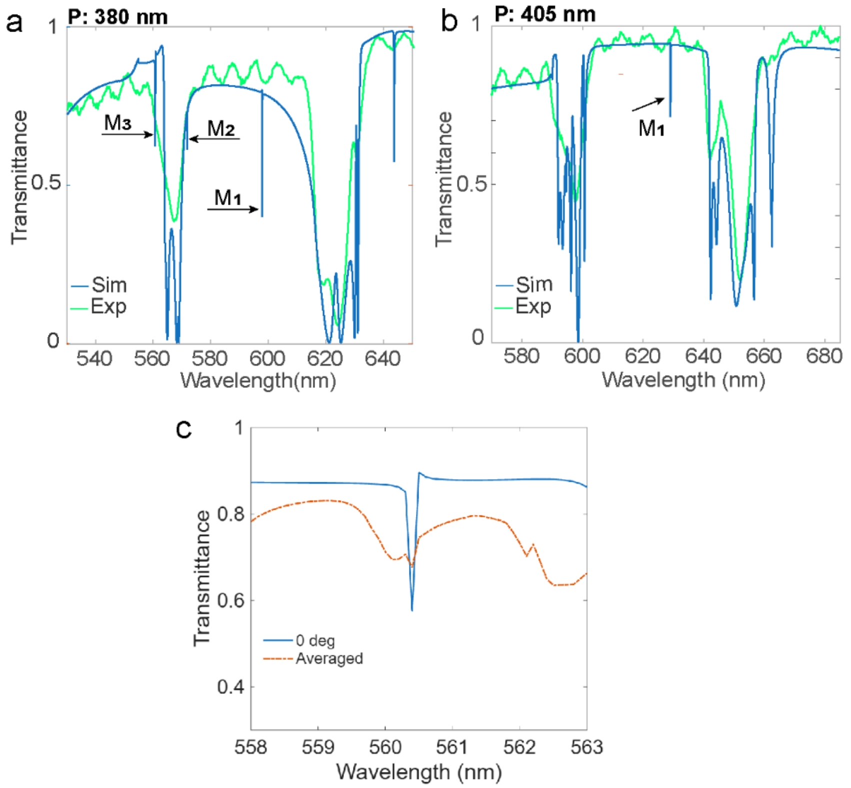

Figure S1. Measured and calculated x-polarized normal-incidence illumination transmission spectra for (a) LMS1 and (b) LMS2. (c) Simulated y-polarized transmission spectra of LMS1 around mode M_3, for normal incidence (blue) and averaged over incidence angles 0–2.5° (orange), illustrating resonance weakening due to angular averaging.

S2: Q-factor of the modes in LMS1

Table S1. Q-factors of the modes in LMS1, extracted from the normal-incidence transmission spectra for x- and y-polarized light using a FWHM-based analysis of the transmission dips.

label	Wavelength (nm)	Q-factor (x-polarized)	Q-factor (y-polarized)
M_1	597	5.91×10^3	5.89×10^3
M_2	571	4.15×10^3	5.19×10^3
M_3	560	5.44×10^3	5.32×10^3

S3: Mode analysis using angular resolved dispersion map

Figure S2a,b show the angular-resolved dispersion maps (wavelength vs emission angle) of the passive metasurface-waveguide system. These maps represent the photonic band structure of the radiative modes accessible in the far field within our experimental collection NA. The radiative region corresponds to the light cone $|\sin\theta| \leq 1$. We overlaid RA diffraction-order curves for the SU8 and SiO_2, calculated from the 2D grating condition for a square lattice of period P:

$nk_0 = |\mathbf{k}_{||} + \mathbf{G}|$, with $k_0 = \frac{2\pi}{\lambda}$ and $\mathbf{G}_{lm} = \frac{2\pi}{P}(l\hat{x} + m\hat{y})$, which for our measured cut (φ= 0) can be written as: $n^2 = (sin\theta + \frac{l\lambda}{P})^2 + (\frac{m\lambda}{P})^2$. Using this relation, we plot the diffraction-order curves, including the diagonal orders (±1, ∓1) in Figure S2a for LMS1 (n_{SU8}= 1.64, n_{sub}= 1.49) and in Figure S2b for LMS2 (n_{SU8}= 1.6, n_{sub}= 1.48) shown as green dashed lines (SU8 side) and white dashed lines (substrate side). In addition, in Figure S2b we overlaid a guided-mode phase-matching reference using the same expression with an effective index n_{eff}= 1.51 (blue dashed line). Note that we do not overlay the axial first order, which passes through Γ; it lies very close to the substrate-side diffraction references because the relevant effective index is similar on the glass side. This produces a weak "duplicate" ridge running nearly parallel to the substrate-side RA lines, visible in both the experiment (Figure 2g) and the high-resolution maps (Figure S2c,d), but it is not cleanly resolved in the full angular range simulation (Figure 2f) due to the coarser sampling.

In Figure S2a (LMS1), M_1 and M_2 coincide with Γ-centered band-edge features of radiative Bloch branches, where the dispersion flattens near $\theta \approx 0$, indicating reduced group velocity and efficient normal-direction outcoupling. These Γ-point features arise from hybridized Bloch resonances in which intrinsically dark meta-atom multipolar modes (quasi-BICs by symmetry breaking) couple to lattice/guided channels. Importantly, the SU8-side diffraction continuum runs

close to the M_1 band-edge region, indicating that lattice diffraction provides an efficient radiation channel once symmetry breaking enables coupling to the radiative continuum.

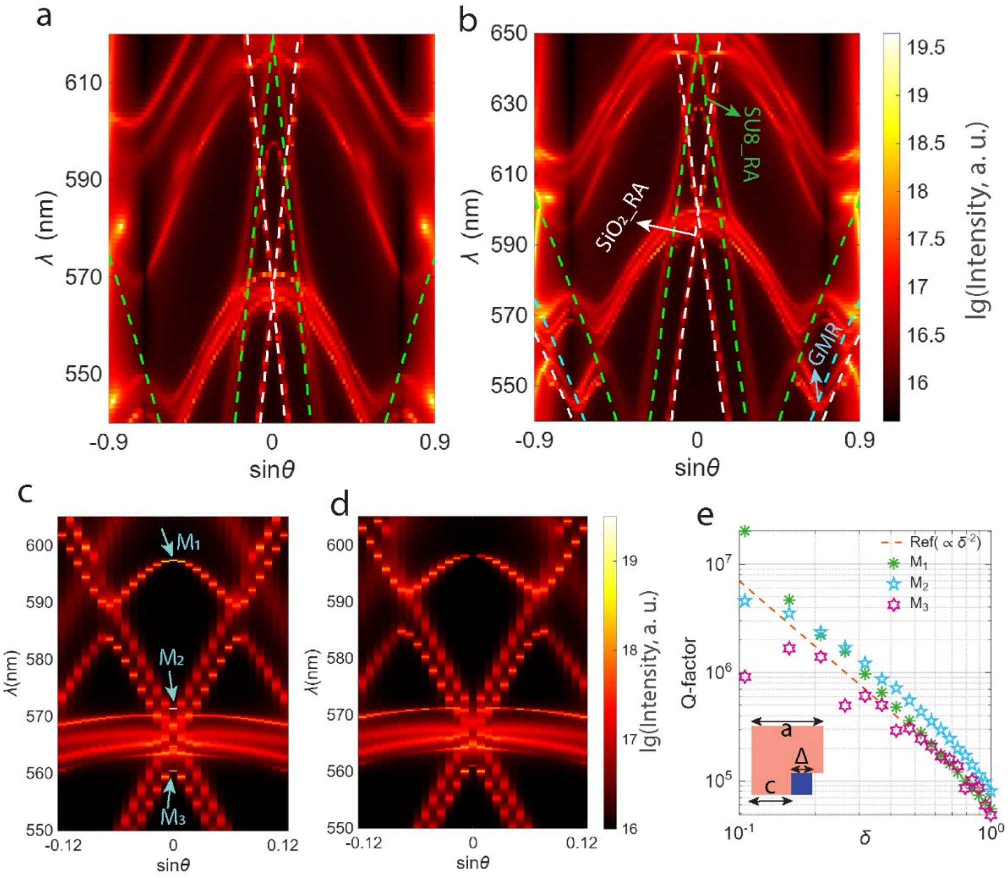

Figure S2. Angular-resolved dispersion maps of the passive metasurface-waveguide system with overlaid diffraction-order (RA) references. (a) LMS1 (*P*=380 nm): SU8-side (green dashed, n_{SU8}= 1.64) and substrate-side (white dashed, n_{sub}= 1.49). (b) LMS2 (*P*=405 nm): SU8-side (green dashed, n_{SU8}= 1.6) and substrate-side (white dashed, n_{sub}= 1.48) references; blue dashed line shows a guided-mode reference (n_{eff}= 1.51). (c,d) Angular-resolved dispersion around Γ-region calculated with a fine wavelength step (0.2 nm) for symmetry-broken and symmetric (notch-free) structures, respectively. (e) Eigenmode Q factors versus asymmetry δ, showing quasi-BIC scaling $Q \propto \delta^{-2}$. A reference $\propto \delta^{-2}$ is shown with an orange dashed line.

By comparison, M_3 (~560 nm) follows a branch near the SiO_2-side RA reference, consistent with a stronger contribution from substrate diffraction channels.

In Figure S2b (LMS2), the two oblique lasing modes appear on distinct dispersive ridges at finite $|\theta|$. M_4 follows the SU8-side RA reference, consistent with a diffraction-coupled (SLR-like) outcoupling channel, whereas M_5 aligns more closely with the guided-mode reference (n_{eff}= 1.51), consistent with a GMR-like channel near $\theta \approx \pm 42°$. While RA reference curves calculated using the bulk SU8 index (n_{SU8}= 1.6) capture the overall dispersion trend, they do not intersect the ~568 nm, ~ $\pm$ 45° point exactly, which is expected because the relevant channels are hybrid Bloch resonances in a finite, asymmetric air/SU8/substrate environment with the metasurface embedded; accordingly, the overlaid references should be viewed as practical guides to identify available outcoupling channels rather than an exact quantitative fit.

To establish the quasi-BIC origin and its connection to Γ-point symmetry, we computed high-resolution dispersion maps in a narrow angular window around Γ ($|\theta| \leq 6^{\circ}$) for both the symmetric (notch-free, Figure S2d) and symmetry-broken geometries (Figure S2c). We then performed a controlled symmetry-breaking study by varying the notch-filling size (Δ) of the L-shaped nanoresonator and defining an asymmetry parameter $\delta = 1 - \frac{\Delta}{a-c}$ where $\delta \rightarrow 0$ corresponds to the symmetric limit (see inset of Figure S2e for definitions). In the symmetric case, M_1 and M_2 are strongly suppressed at Γ, while M_3 remains weakly visible, indicating greatly reduced (but not fully eliminated) radiative coupling in the symmetric configuration; all three modes become notably brighter under intentional symmetry breaking. Consistently, the eigenfrequency-extracted Q-factors of M_1 and M_2 follow the expected quasi-BIC scaling Q $\propto \delta^{-2}$ in the perturbative regime (Ref [22] of the manuscript and Figure S2e), characteristic of Γ-point symmetry protection. M_3

also shows quasi-BIC-like enhancement with decreasing δ, but its Q is systematically lower, consistent with an additional δ-independent leakage channel enabled by coupling to substrate diffraction (RA/SLR-related) continuum.

S4: Calculated normalized near-field intensity profiles and mode-overlap analysis

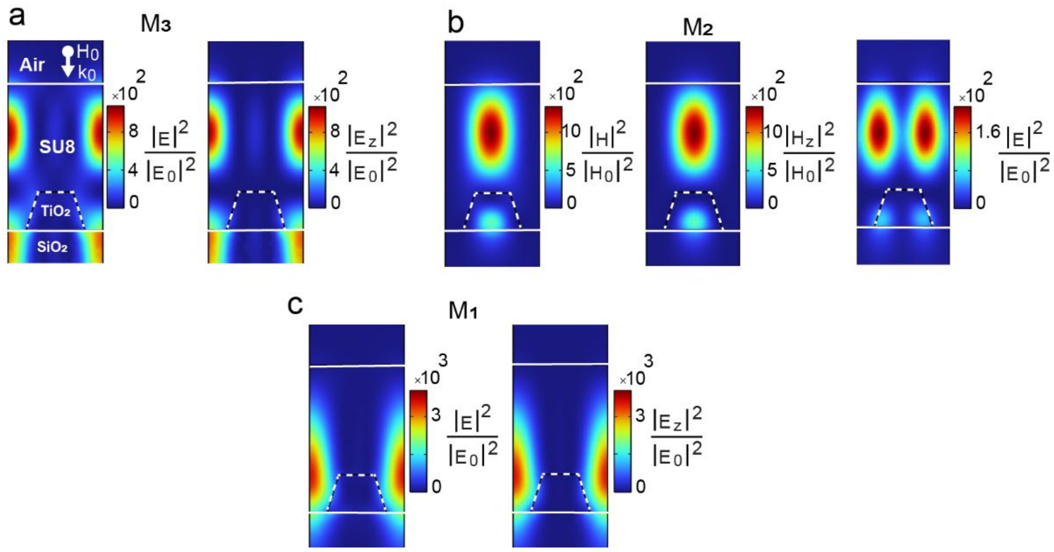

Figure S3. Calculated normalized near-field intensity $\frac{|E(H)|^2}{|E_0(H_0)|^2}$ profiles of LMS1 in the yz cross-section through the center of the nanoresonator for y-polarized normal incidence illumination and contribution of the dominant field components $E_z(H_z)$ to the observed enhancement at (a) 560 nm (M3), (b) 570 nm (M2), and (c) 600 nm (M1). Mode profiles are normalized with respect to the corresponding intensity of the incident plane wave. Dashed white lines denote the nanoresonator outlines.

Calculated normalized near-field intensity profiles of LMS1 for modes M_1–M_3 are shown in Figure S3. While all three modes reside in the same SU8 gain layer, their field distributions differ substantially, consistent with the distinct mode character discussed in the main text. To quantify

the degree of spatial competition in the gain medium, we compute a mode-mode overlap factor within the SU8 volume V using COMSOL Multiphysics (Wave Optics Module, ewfd).

Defining $I_m(\boldsymbol{r}) = |\boldsymbol{E}(\boldsymbol{r}, \lambda_m)|^2$, the overlap between modes m and n is evaluated as $X_{mn} = \frac{\int_V I_m(\boldsymbol{r}) \times I_n(\boldsymbol{r})\, dV}{\sqrt{\int_V I_m^2(\boldsymbol{r}) dV \times \int_V I_n^2(\boldsymbol{r}) dV}}$, where the integrals are performed by volume integration over the SU8 domain. For LMS1 we obtain $X_{12} = 0.24$, $X_{23} = 0.49$, and $X_{13} = 0.57$, indicating weak-to-moderate spatial overlap within the gain region. This nonzero overlap is expected because the modes share the same gain layer; however, the values confirm that the modes are not fully overlapping, and each draws gain preferentially from different regions of the SU8. Together with the substantial spectral separation of M_1–M_3 across the Rh6G gain band, these results support limited mutual gain depletion and help explain the observed stable simultaneous multimode lasing.

S5: Multipole decomposition of the modes

To quantify the contributions of different multipoles to the optical response, we performed a multipole decomposition of the scattered displacement currents density inside the nanoresonators, following the approach of Grahn et al.[1,2] All simulations were carried out in COMSOL Multiphysics for a single unit cell of the metasurface using periodic boundary conditions and a normal incident plane wave. In the first step, we computed the background solution (substrate and superstrate without the nanoresonator). In the second step, we treated the nanoresonator as the scatterer and used COMSOL's scattered-field formulation to obtain the scattered displacement current density inside the TiO_2 nanoresonator. These numerically obtained currents were then projected onto the electric and magnetic multipole moments[1] and used to classify the modes in terms of electric and magnetic dipole and quadrupole contributions. The results for a *y*-polarized (as a representative example), normal incident plane wave are presented in Figure S4. The modes

can be classified in terms of associated Legendre polynomials[3] $P_l^m(x)$, where $l = 1,\ m = 0,\ \pm 1$ stand for the dipolar modes and $l = 2,\ m = 0,\ \pm 1,\ \pm 2$, correspond to quadrupolar modes. We can see that M_3 (560 nm) and M_1 (597 nm) are dominated by an in-plane MQ ($l = 2,\ m = \pm 2$), and M_2 (570 nm) is governed by an out-of-plane MD mode ($l = 1,\ m = 0$).

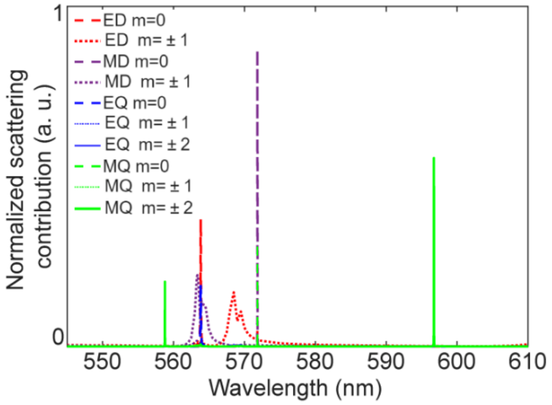

Figure S4. Multipolar decomposition of the modes for y-polarized normal incidence illumination.

S6: Fabrication process of the metasurfaces

Metasurface samples were fabricated using an electron beam lithography (EBL)-based process. The procedure began with cleaning a fused silica substrate (Siegert) in a cleaning system (OPTIwet SB30). A 156 nm TiO_2 layer was then deposited onto the substrate via ion beam deposition (Oxford Instruments, Ionfab 300), followed by the deposition of a ~30 nm chromium (Cr) layer using the same system. This Cr layer served as a hard mask for subsequent etching. Next, a negative electron-beam resist (ma-N2401) was spin-coated onto the sample and patterned with a variable-shaped EBL system (Vistec SB350). After development in OPD4262, the exposed Cr regions were removed by ion beam etching (Oxford Instruments, Ionfab 300). The resulting Cr pattern acted as a hard mask for transferring the design into the TiO_2 layer via reactive ion etching in CF_4 gas at a flow rate of 30 SCCM. Finally, the remaining resist was stripped with acetone, and the residual Cr was removed using a chromium etchant.

S7: Gain medium preparation

We prepared the solution by dissolving 10 mg of Rh6G dye powder in 2 mL of cyclopentanone and subsequently mixing it with 1 mL of the negative-tone photoresist SU8-2005. The mixture was then ultrasonicated for 10 minutes to ensure homogeneity. The resulting solution contained Rh6G at a concentration of 0.325 wt%. The fabricated metasurfaces were spin-coated with this solution for 5 s at 500 rpm, followed by 60 s at 6000 rpm, yielding a film thickness of 570 nm. The coated samples were then baked at 95℃ for 2 minutes to remove excess cyclopentanone. The SU8 thickness of 570 nm was selected through parametric sweeps in our FEM simulations to maximize mode-gain overlap and to align the lowest-order GMRs with the Rh6G gain bandwidth. Thinner films reduce field confinement and the mode-gain interaction volume, while significantly thicker films can support higher-order GMRs and weaken coupling to the metasurface lattice, thereby compromising efficient feedback and device compactness. The Rh6G concentration (0.325 wt% in SU8) was selected empirically through iterative testing as the highest value that remained fully dissolved in the cyclopentanone/SU8 without noticeable precipitation or saturation during preparation, produced a uniform and optically bright film after spin coating and baking, and preserved stable emission and lasing behavior over the timescale required for optical measurements. Under our pumping conditions (low repetition rate of 1 Hz and moderate fluence), we did not observe noticeable pump-induced photobleaching during repeated measurements across multiple sessions. However, longer ambient storage could lead to gradual degradation of the dyes over several days, consistent with the oxygen-related photobleaching effects. Low dye concentrations are generally expected to reduce the available optical gain and increase lasing thresholds, while excessively high concentrations can introduce adverse effects such as self-

absorption, concentration quenching, and reduced net gain due to enhanced loss, as widely reported for dye-doped polymer thin films.[4]

S8: Instrument-response-corrected linewidth extraction

The measured linewidths of the lasing peaks are limited by the finite spectral resolution of the spectrometer. To retrieve the intrinsic linewidth of the emission lines, we performed an instrument-response-corrected analysis based on forward-convolution fitting.[5] The procedure consists of three steps. (1) Mode isolation: for each spectrum, a fixed wavelength window around the lasing line was selected and the broadband Rh6G fluorescence background within this window was removed using a local baseline subtraction (linear fit to the spectral edges), yielding an isolated narrow peak; (2) instrument lineshape (ILS) calibration: the spectrometer response was characterized experimentally using a narrow-linewidth 532 nm pulsed laser recorded with the same optical configuration and spectrometer settings as used for the metasurface measurements (Teem Photonics STG-03E-130; datasheet linewidth 0.8 pm). After baseline subtraction and normalization to unit area, the measured ILS exhibited a FWHM of ≈ 1.67 nm. Because this is comparable to the apparent linewidth of the lasing peaks in the raw spectra (≈ 1.5 nm) an explicit ILS-based correction can help to avoid instrument-limited linewidth estimates; and (3) intrinsic linewidth fitting: using forward-modeled fitting, a Lorentzian lineshape representing the lasing mode was convolved with the measured ILS (shifted to the corresponding emission wavelength) and fitted to the isolated peak, yielding the intrinsic linewidth for each pump energy. This approach enables the extraction of sub-resolution linewidths. Using this method, the intrinsic linewidth of the lasing peaks is found ≈ 0.26 nm at and above threshold.

S9: S-curves on linear–linear scale

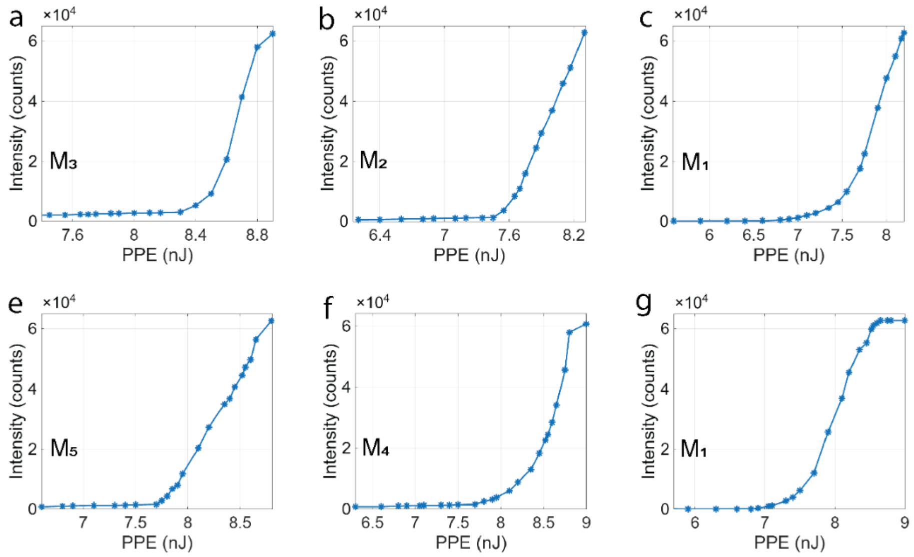

Figure S5. Maximum output intensity at different PPEs (S-curve) on linear–linear scale for (a–c) LMS1, and (e–g) LMS2.

S10: Experimental setup

The sample was pumped with a 532 nm pulsed laser (Teem Photonics STG-03E-130; 0.5 ns, maximum 3 μJ per pulse). A collimated beam passed an adjustable square aperture (≤12 mm × 12 mm) which was imaged onto the selected metasurface to provide uniform illumination within a 140 × 140 μm^2 region. Emission was collected with a 60×/0.9 NA objective (Olympus UPLFLN) and directed either to a spectrometer (Ocean Insight FLAME-S-VIS-NIR-ES; 1.34 nm FWHM) for spectral measurements or to an EMCCD (Andor iXon Ultra 897) for back-focal-plane (BFP) imaging (Figure S6). For power-dependent spectra, the pump pulse energy was varied from 0–9 nJ using a variable neutral-density filter. For BFP and momentum-resolved spectroscopy, the BFP of the objective was imaged by a 4f system (two 200 mm achromatic lenses, Thorlabs AC254-200-B) onto the EMCCD or the entrance slit of an imaging spectrometer (Andor Kymera 328i),

respectively. Acquisition settings were: below threshold, 500 Hz repetition rate with 2 s integration to collect sufficient photons in the spontaneous emission regime; at/above threshold, 1 Hz with 1 s integration time to avoid Rh6G dye bleaching. Band-pass filters at 550 nm, 560 nm, 570 nm and 600 nm (10 nm FWHM) were inserted in the detection path to select the emission associated with specific modes of the hybrid system.

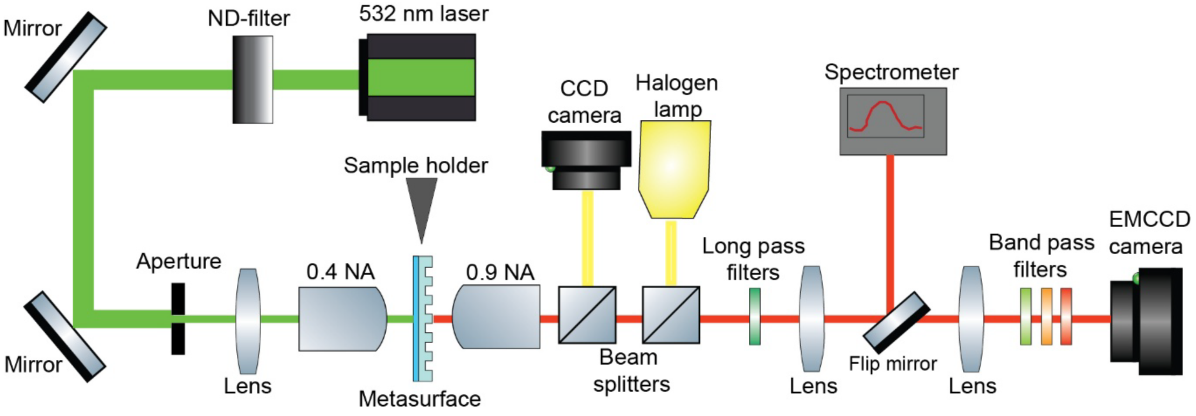

Figure S6. Sketch of the experimental setup used for lasing characterization.

S11: Identifying lasing modes of LMS1 via eigenfrequency analysis

We performed an eigenfrequency analysis of the periodic metasurface, using the finite element method (FEM) implemented in COMSOL Multiphysics, under normal-incidence plane wave illumination and applying periodic boundary conditions to confine the computational domain to a single unit cell. To mimic small-signal gain, the SU8 layer containing Rh6G on top of the metasurface was modeled with a refractive index $n = n' + i\kappa$, where n' is the real part of the refractive index of SU8 and $\kappa < 0$, $|\kappa| \ll 1$ is an artificial modification, introduced only in the simulation, to identify lasing-prone modes.

With the field time dependence of $\boldsymbol{E}(\boldsymbol{r}, t) \propto e^{-iwt}$, the computed eigenfrequencies are $\omega = \omega' + i\omega''$, where $\omega'' < 0$ indicates temporal growth (net gain), whereas $\omega \geq 0$ indicates decay

(net loss). For $\kappa = -6 \times 10^{-5}$ our calculations yield four modes with $\omega'' < 0$, three of which lase in the optical experiments, while all other computed modes have $\omega \geq 0$. Note, the absolute values of ω'' depend on the chosen κ, so only the sign and relative trends are physically meaningful. Table S2 summarizes the calculated results.

Table S2. Calculated eigenfrequencies of LMS1 with a period of 380 nm, for normal incidence plane wave illumination.

**Eigenfrequency (THz)**	**Wavelength (nm)**
501.88-0.0094i	597.33 (M_1)
524.63-0.013i	571.43 (M_2)
530.8-0.0073i	564.80
534.95-0.0077i	560.41 (M_3)

S12: Polarization-dependent emission spectra

We showed for the transmission spectra of the passive structure (Figure 1c,d, and Figure S1a,b) that sharp resonances, including M_1–M_3, appear under both x and y polarizations, with stronger near-field enhancement for y polarization. Similarly, lasing occurs for both x- and y-polarizations for these three modes; however, in contrast to transmission, the lasing emission exhibits a clear polarization preference for each mode. As evident in the polarization-resolved lasing emission spectra of LMS1 (Figure S7), M_3 (560 nm) shows nearly balanced output with a slight preference for x-polarization, M_2 (570 nm) lases most strongly under y-polarization, and M_1 (600 nm) preferentially emits with x-polarization. The partial mismatch between passive enhancement and lasing output highlights that lasing does not simply follow the strongest incoupling channel.

Instead, the radiative pathway with the most favorable gain-to-loss balance (lowest threshold) dominates, leading to complementary polarization preferences for the three modes.

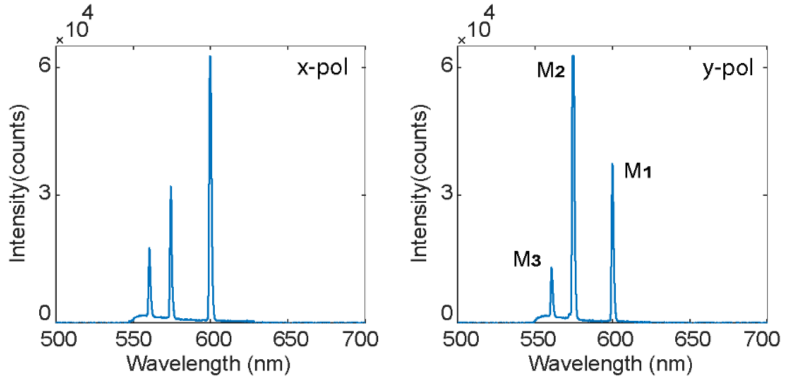

Figure S7. Polarization-resolved lasing emission spectra of LMS1 showing x-polarized-dominated lasing for M_1 (600 nm) and M_3 (560 nm), and y-polarized-dominated lasing for M_2 (570 nm).

S13: Calculation of the angle-resolved emission spectra

We simulated angle-resolved spontaneous emission spectra of the metasurface coated with an SU8 layer containing randomly oriented, homogeneously distributed point dipole emitters using COMSOL Multiphysics, based on the reciprocity principle (Ref [37] of the manuscript). This approach allows the computational domain to be reduced to a single elementary unit cell with periodic boundary conditions, while still accounting for the isotropic orientation of the dipoles. Within this approach, the power $P(\theta, \varphi; \boldsymbol{r}_i)$ radiated into the far field in the direction θ (polar angle) and φ (azimuthal angle) with TE (TM) polarization by a randomly fluctuating dipole $\boldsymbol{p_i}$ (on average isotropically oriented) placed at position $\boldsymbol{r}_i$ on the metasurface is proportional to $|\boldsymbol{E}_{local}(\boldsymbol{r}_i)|^2$. Here, $\boldsymbol{E}_{local}$ denotes the local electric field of the metasurface at the position of the dipole excited by a TE- (TM-) polarized plane wave incident from direction (θ, φ) at the wavelength corresponding to the electric dipole (ED) transition. The emission intensity of the ED

in the direction (θ, φ) was then calculated as, $P(\theta, \varphi) \propto \langle \frac{\sum_{TE,TM} |\boldsymbol{E}_{local}(\theta,\varphi,\boldsymbol{r}_i)|^2}{\boldsymbol{E}_0} \rangle$ where $P(\theta, \varphi)$ is the emitted power along the direction (θ, φ) at wavelengths of interest, and $\boldsymbol{E}_0$ the electric field amplitude of the plane wave incident onto the structure along the same direction. The notation $\langle \ \rangle$ indicates spatial averaging over the active volume of the sample containing the emitters.

S14: Calculated normalized near-field intensity profiles of modes at an oblique angle

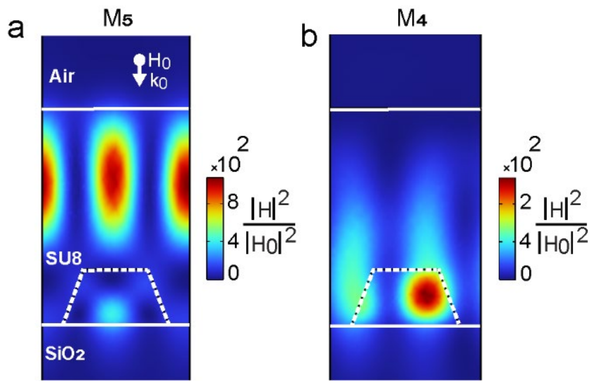

Figure S8. Calculated near-field intensity profiles of LMS2 in the yz cross-section through the center of the nanoresonator for TE-polarized illumination for a) M_5 at $\theta = 42^{\circ}, \varphi = 0, \lambda \approx 548$ nm, and b) M_4 at $\theta = 45.3^{\circ}, \varphi = 0, \lambda \approx 568$ nm, showing that M_5 and M_4 are dominated by a GMR and an SU8-side SLR, respectively. Mode profiles are normalized with respect to the intensity of the incident plane wave. Dashed white lines indicate the nanoresonator outlines.

S15: Angular resolved emission spectra of lasing metasurfaces with the longest and shortest lasing wavelengths for Rh6G dyes

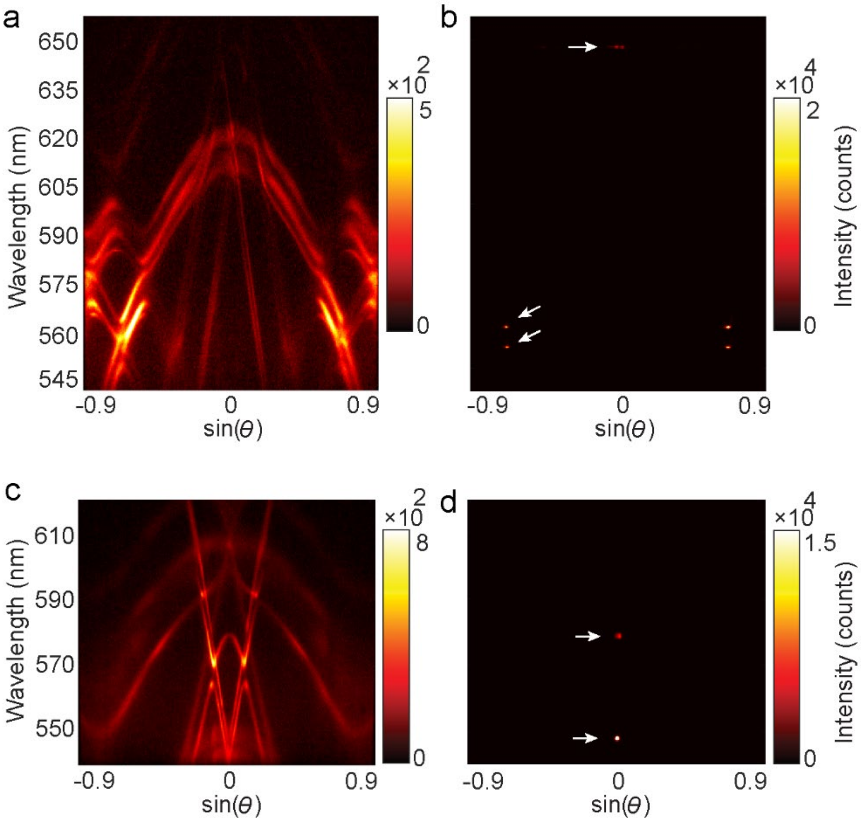

Figure S9. Measured angle-resolved emission spectra of metasurfaces coated with Rh6G-doped SU8 and having periods of (a,b) 415 nm and (c,d) 360 nm, shown (a,c) below and (b,d) above threshold. Near-normal direction lasing at 648 nm is observed for the metasurface with *P*=415 nm, while lasing in the normal direction at 548 nm occurs for the metasurface with *P*=360 nm, representing the longest and shortest lasing wavelengths among the investigated systems.

From the below threshold momentum-resolved spectra in Figure S9a,c, and the period-dependent lasing map in Figure 4a, we associate the long- and short-wavelength limits of our platform with modes M_1 and M_2, respectively. While lasing near 630 nm occurs where Rh6G absorption is negligible, absorption at 548 nm remains non-negligible. Nevertheless, the 548 nm lasing

corresponds to mode M_2 (Figure S9c,d), a symmetry-broken quasi-BIC with dominant MD_z character hybridized with a TE_1-like guided-mode channel, providing high feedback and reduced radiative/scattering loss such that the available Rh6G gain can overcome the residual absorption tail.

S16: Angular resolved emission spectra of the lasing metasurface with four lasing peaks

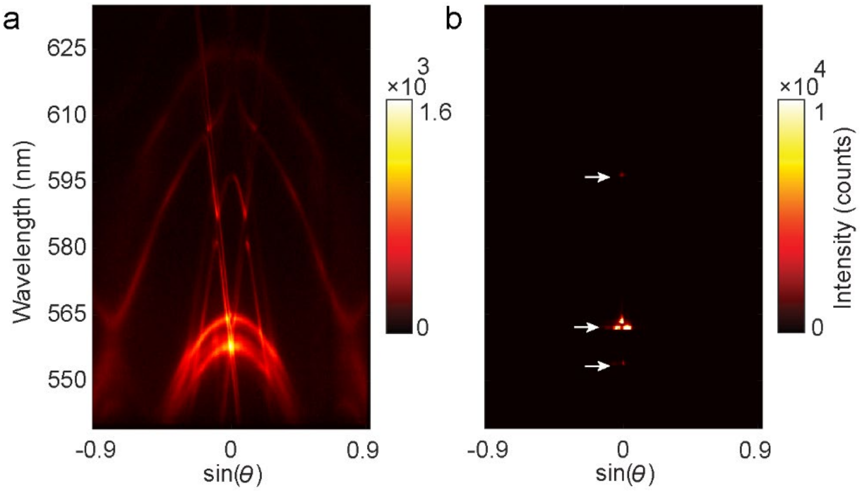

Figure S10. Measured angular resolved emission spectra of the metasurface laser with lateral dimensions 10% larger than the other metasurfaces reported in this work, and P=370 nm, (a) below, and (b) above threshold. We observe four lasing peaks at 553 nm, 561 nm, 563 nm, and 596 nm.

S17: Amplified spontaneous emission (ASE) or lasing

ASE can show partial laser-like behavior (spectral narrowing, polarization, and directionality), but it typically exhibits a substantially broader linewidth (~10 nm), and the resonator does not strongly define the emission.[6] As a quantitative benchmark for Rh6G, Rau et al.[7] report narrowing from a fluorescence bandwidth of ≈ 39 nm to an ASE linewidth of ≈ 16 nm, and He et al.[8] report linewidths narrowing to ≈ 6–7 nm in high-gain capillary ASE/random-lasing regimes. In our

measurements, the raw lasing peaks have FWHMs of approximately 1.5 nm, limited by the spectrometer response; using the measured ILS and a forward-convolution fit (Section S8), we extract intrinsic linewidths of approximately 0.26 nm, making an ASE-based interpretation unlikely. In addition, the emission does not merely become directional; it undergoes an abrupt redistribution in momentum space: below threshold, it is distributed along dispersive diffraction features, whereas above threshold, it collapses into sharply localized Γ-point (LMS1) or fixed-angle $\pm\theta$ (LMS2) features, consistent with cavity-eigenmode selection and oscillation rather than broadband ASE outcoupling. Finally, the transition from ASE-dominated emission to lasing is quantified by mode-resolved thresholds extracted from the S-curves. For instance, for modes in LMS1, the thresholds are: M_1= 6.9 nJ, M_2= 7.5 nJ, and M_3= 8.4 nJ. These independent thresholds, together with the momentum-space condensation and sub-instrument intrinsic linewidths, support lasing rather than a cavity-filtered ASE.

REFERENCES

(1) Grahn, P.; Shevchenko, A.; Kaivola, M. Electromagnetic multipole theory for optical nanomaterials. *New J. Phys.* **2012**, *14* (9), 093033. https://doi.org/10.1088/1367-2630/14/9/093033

(2) Vaskin, A. Controlling Light Emission by Dielectric Metasurfaces. Ph.D. Thesis, Friedrich-Schiller-Universität Jena, Jena, 2020. https://doi.org/10.22032/dbt.47340

(3) Jackson, J. D. Classical Electrodynamics; Wiley: Hoboken, NJ, 2012.

(4) Vogelbacher, F.; Zhou, X.; Huang, J.; Li, M.; Jiang, K.-J.; Song, Y.; Unterrainer, K.; Hainberger, R. Material Gain Concentration Quenching in Organic Dye-Doped Polymer Thin Films. *Opt. Mater. Express* **2019**, *9* (3), 1208–1218. https://doi.org/10.1364/OME.9.001208

(5) Aster, R. C.; Borchers, B.; Thurber, C. H. *Parameter Estimation and Inverse Problems*, 3rd ed.; Elsevier: Amsterdam, 2018. https://doi.org/10.1016/C2015-0-02458-3

(6) Samuel, I. D. W.; Namdas, E. B.; Turnbull, G. A. How to Recognize Lasing. *Nat. Photonics* **2009**, *3* (10), 546–549. https://doi.org/10.1038/nphoton.2009.173

(7) Rau, I.; Szukalski, A.; Sznitko, L.; Miniewicz, A.; Bartkiewicz, S.; Kajzar, F.; Sahraoui, B.; Mysliwiec, J. Amplified Spontaneous Emission of Rhodamine 6G Embedded in Pure Deoxyribonucleic Acid. *Appl. Phys. Lett.* **2012**, *101* (17), 171113. https://doi.org/10.1063/1.4764535

(8) He, H.; Russell-Hill, P.; Blau, W. J.; Kislyakov, I. M.; Rosanov, N. N.; Dong, N.; Wang, J. Amplified Spontaneous Emission and Random Laser Generation in Capillaries Doped with Rhodamine 6G–Microspheres. *Appl. Opt.* **2025**, *64* (12), 3383–3390. https://doi.org/10.1364/AO.558305
